# Enhanced efficiency of zinc oxide hydroxyapatite nanocomposite in photodegradation of methylene blue, ciprofloxacin, and in wastewater treatment

**DOI:** 10.1038/s41598-025-14310-7

**Published:** 2025-08-29

**Authors:** K. Manikandan, N. Dinesh Kumar, R. Velmurugan, M. Gokulnath, S. Parvathy, Manikandan Ayyar, M. Swaminathan, P. Prabhu, Abdullah M. S. Alhuthali, Magda H. Abdellattif, Rajesh Haldhar, M. Khalid Hossain

**Affiliations:** 1 Department of Chemistry, SRM Institute of Science and Technology, Ramapuram Campus, Chennai, Tamil Nadu India; 2 Department of Chemistry, Erode Sengunthar Engineering College, Erode, 638057 Tamil Nadu India; 3 Department of Chemistry, Ramakrishna Mission Vivekananda College, Chennai, 600028 India; 4 Department of Chemistry, Government Arts College (Autonomous), Salem, 636007 Tamil Nadu India; 5 Department of Chemistry, Karpagam Academy of Higher Education, Coimbatore, 641 021 Tamil Nadu India; 6 Centre for Material Chemistry, Karpagam Academy of Higher Education, Coimbatore, 641021 Tamil Nadu India; 7 Department of Chemistry, Kalasalingam Academy of Research and Education, Krishnan Koil, 626126 Tamil Nadu India; 8 Research and Innovation Cell, Rayat Bahra University, Mohali, Punjab 140301 India; 9 Department of Mechanical Engineering, Mattu University, 318, Mettu, Ethiopia; 10 Department of Physics, College of Sciences, Taif University, P.O. Box 11099, 21944 Taif, Saudi Arabia; 11 Department of Chemistry, College of Sciences, University College of Taraba, Taif University, P.O. Box 11099, 21944 Taif, Saudi Arabia; 12 School of Chemical Engineering, Yeungnam University, Gyeongsan, 38541 Republic of Korea; 13 Institute of Electronics, Atomic Energy Research Establishment, Bangladesh Atomic Energy Commission, Dhaka, 1349 Bangladesh; 14 Department of Advanced Energy Engineering Science, Interdisciplinary Graduate School of Engineering Sciences, Kyushu University, Fukuoka, 816- 8580 Japan

**Keywords:** Zinc oxide, Photocatalysis, Hydroxyapatite, Methylene blue, Wastewater treatment, Ciprofloxacin, Chemistry, Engineering, Physics

## Abstract

In this study, we report the improved photocatalytic performance of Hydroxyapatite-incorporated Zinc Oxide Nanocomposite (ZnO@HAp), synthesized using a simple precipitation method. The catalyst was characterized using powder X-ray diffraction (XRD), Fourier transform infrared spectroscopic analysis (FT-IR), Ultraviolet–Visible Diffuse Reflectance Spectroscopy (UV-DRS), Scanning Electron Microscopy (SEM) with energy-dispersive X-ray spectroscopy (EDX), and X-ray photoelectron spectroscopy (XPS). The formation of ZnO@HAp was confirmed by XRD, FT-IR, EDAX, and XPS. SEM images of the ZnO@HAp reveal that the particles were agglomerated, and spherical-shaped ZnO particles were present on the surface of HAp.The presence of particles in various forms, such as spherical and hexagonal structures, is visible on the surface of pure HAp in the TEM images of the material. The XPS analysis confirmed the presence and chemical states of essential elements (Zn, O, Ca, and P) in the ZnO@HAp composite. The photocatalytic efficiency of the synthesized composite was evaluated by the degradation of organic pollutants, including methylene blue and ciprofloxacin, as well as in domestic sludge water treatment. The removal efficiencies of 96.6% for methylene blue (30 min), 62% for ciprofloxacin (150 min), and 88% COD reduction in domestic sludge were observed. This composite gives remarkable degradation performance due to its stability and good photocatalytic properties. The results obtained from the prepared ZnO@HAp composite, compared to ZnO and pure HAp, showed higher photocatalytic efficiency with long-term stability, without any changes in the ZnO@HAp characteristics after five consecutive runs. The enhanced properties of the ZnO@HAp composite make it a promising candidate for environmental remediation applications, offering an effective solution for pollutant degradation in wastewater treatment.

**Supplementary Information:**

The online version contains supplementary material available at 10.1038/s41598-025-14310-7.

## Introduction

One of the most vital resources for life is water, yet a variety of organic and inorganic contaminants, including dyes, pesticides, and antibiotics, contaminate it. Polluted water usage leads to a wide range of health issues in humans and animals. Dyes present in the water can create severe health problems, such as eye burning, stomach stimulation, and allergy symptoms, including nausea and diarrhea^1^. Textile industry effluent is found to have a high concentration of methylene blue (MB) dye, as it is a widely used azo dye in the textile industry, and its discharge into a water body has a profound impact on the aquatic environment. Therefore, it is crucial to treat the wastewater contaminated with MB^2^. Antibiotics in the environment promote the spread of antibiotic-resistant genes and bacteria, resulting in adverse effects on aquatic life and human health^3^. Ciprofloxacin, a potent fluoroquinolone antibiotic, inhibits microbial growth by blocking the replication of cells^4^.

Various water and wastewater treatment processes have been employed, and some of these are expensive and not commercially viable for large-scale treatment. For example, the adsorption process simply transfers pollutants from one phase into another without completely removing them from the environment. AOPs, or advanced oxidation processes, have been developed to address the growing demand for efficient wastewater treatment. Wastewater contaminants are totally destroyed by the hydroxyl radical, a potent oxidizing agent produced by AOPs. Among the AOPs, heterogeneous photocatalysis using semiconductor oxides has been explicitly used for the degradation of toxic chemicals^5,6^.

Hydroxyapatite (HAp) is a well-known synthetic biocompatible and porous material that has been widely used in medical field applications, such as bone implants or as coatings on prosthetics^7^. Hydroxyapatite [Ca_10_(PO_4_)_6_(OH)_2_] combined with metal oxides yields good light absorption and enhanced photodegradation properties^8–10^. Hydroxyapatite has a high capacity for adsorption towards certain organic pollutants, such as dyes and antibiotics, which allows for an increase in the density of organic substrate population in the vicinity of the ZnO photocatalyst. HAp with increased adsorption can capture more electron-hole pairs to react with adsorbed O_2_ and H_2_O molecules on the surface of the ZnO/HAp, generating abundant ·OH radicals for decomposing the dye/ciprofloxacin adsorbed on the surface of the catalyst^11^.

Nano-structures of metal oxides have important applications in various fields due to their different properties of oxides. Zinc Oxide is an important metal oxide that can be easily synthesized and acts as an excellent photocatalyst. ZnO nanoparticle is widely used for industrial, chemical, physical, and medical applications due to its unique morphology and properties^12,13^. ZnO has a crystalline wurtzite structure and a substantial band gap (3.37 ​eV at room temperature). The photocatalytic performance of ZnO had been enhanced by loading with good support materials^14–16^. ZnO can act as a good photocatalyst to degrade the hazardous dyes found in aqueous environments. Nano-ZnO has been synthesized by a simple precipitation method^17^. Hydroxyapatite (HAp)as a support material enhanced the performance of metal oxides in photocatalytic activity. Comparatively, zinc oxide is an excellent material to be composited with HAp for high photo-sensitivity. ZnO is considered an efficient catalyst for the photodegradation of surrounding pollutants due to a rapid electron transfer. Zinc oxide nanoparticles associated with highly porous material nano-Hydroxyapatite (HAp) may increase the photocatalytic property of ZnO.

Rahman et al. employed a solid-state combustion approach to develop the biomaterial-based photocatalytic composite hydroxyapatite–TiO_2_–ZnO (HAp-TiO_2_–ZnO) from HAp prepared using eggshell and di-ammonium hydrogen phosphate, and they photodegraded methylene blue to test its effectiveness^18^. The porous ZnO-hydroxyapatite nanocomposite’s ability to degrade the model dye methylene blue was examined. When calcined at 500 °C, ZnO@HAp is found to break down the contaminants more quickly than ZnO. Jin-Chung Sin et al. proposed a simple, environmentally friendly process for synthesizing ZnO@HAp composite using *Punica granatum* peel extract-derived cauliflower-like ZnO decorated with HAp derived from bovine bone. In addition to studying bacterial regrowth trials to ascertain the viability of as-synthesized materials in photocatalytic disinfection, it demonstrated the most potent antibacterial activity against *Enterococcus faecalis*. They also discovered that these compounds are safe for use in water disinfection^19^. Wet impregnation was used by Tanji et al. to create zinc oxide-hydroxyapatite composites. ZnO immobilization was supported by hydroxyapatite (HAp), which was also utilized as a photocatalyst to photodegrade two harmful pollutants, including rhodamine B molecules and caffeine. They discovered that, when exposed to UV light, the ZnO-HAp had better photocatalytic activity than ZnO alone^20^. The highly porous and adsorbent properties of HAp with ZnO significantly improved the photodegradation of antibiotics in a water medium and efficiently trapped the by-product^21^. Therefore, the catalysts prepared by simple and cost-effective methods, exhibited much better photocatalytic performance when compared to ZnO and HAp. Consequently, we firmly believe that a simple methodology will facilitate the development of efficient photocatalysts for future environmental applications.

Therefore, in this work, we report a nano ZnO-loaded HAp nanocomposite prepared by a simple precipitation method and its photocatalytic activity. The photocatalytic activity of the prepared material demonstrated better performance compared to pure ZnO under optimized experimental conditions, resulting in high removal efficiencies of methylene blue and ciprofloxacin, as well as in the treatment of domestic sludge wastewater under UV light illumination. This cost-effective composite gives remarkable degradation properties due to its stable and good photocatalytic properties.

## Experimental

### Materials

Zinc acetate dihydrate, sodium hydroxide, dipotassium hydrogen phosphate trihydrate, calcium Chloride, and methylene blue dye were purchased from SD Fine Chemicals, and the ciprofloxacin drug was supplied by Hi-media. The deionized water was used for dilution. All the chemicals were used without further purification.

### Synthesis of hydroxyapatite (HAp)

Dipotassium hydrogen phosphate (K_2_HPO_4_·3H_2_O) was prepared in a 0.1 M solution and blended with a 0.1 M sodium hydroxide (NaOH) solution, maintaining a pH of 10.5. Calcium chloride (CaCl_2_) was added dropwise to the aforesaid mixture while being continuously stirred after being separately dissolved in 60 mL of deionized water. The milky white precipitate obtained was refluxed at 90 °C for 2 h. After cooling, centrifugation was used to separate the precipitate, which was then cleaned with deionized water. The hydroxyapatite gel-like mixture was then dried overnight at 80 °C in an oven^22^.

### Preparation of zinc oxide nanoparticles

The precipitation process was used to fabricate the ZnO nanoparticles. Equimolar concentrated (0.1 M) solutions of zinc acetate and potassium hydroxide were used. Initially, 100 mL of 0.1 M Zn(Ac)_2_·2H_2_O solution was added dropwise to 100 mL of 0.1 M NaOH solution contained in a 400 mL beaker, with constant vigorous stirring for approximately 10 min at room temperature, until a milky mixture of Zn(OH)_2_ was obtained. This mixture was kept in an autoclave and heated for 8 h at 150 °C. The white sediment was centrifuged and washed with distilled water and ethanol. Finally, the zinc hydroxide was dried in an oven at 90 °C overnight and calcined in a muffle furnace at 500 °C for 2 h^23^.

### Synthesis of zno@hap nanocomposite

**Scheme 1:**
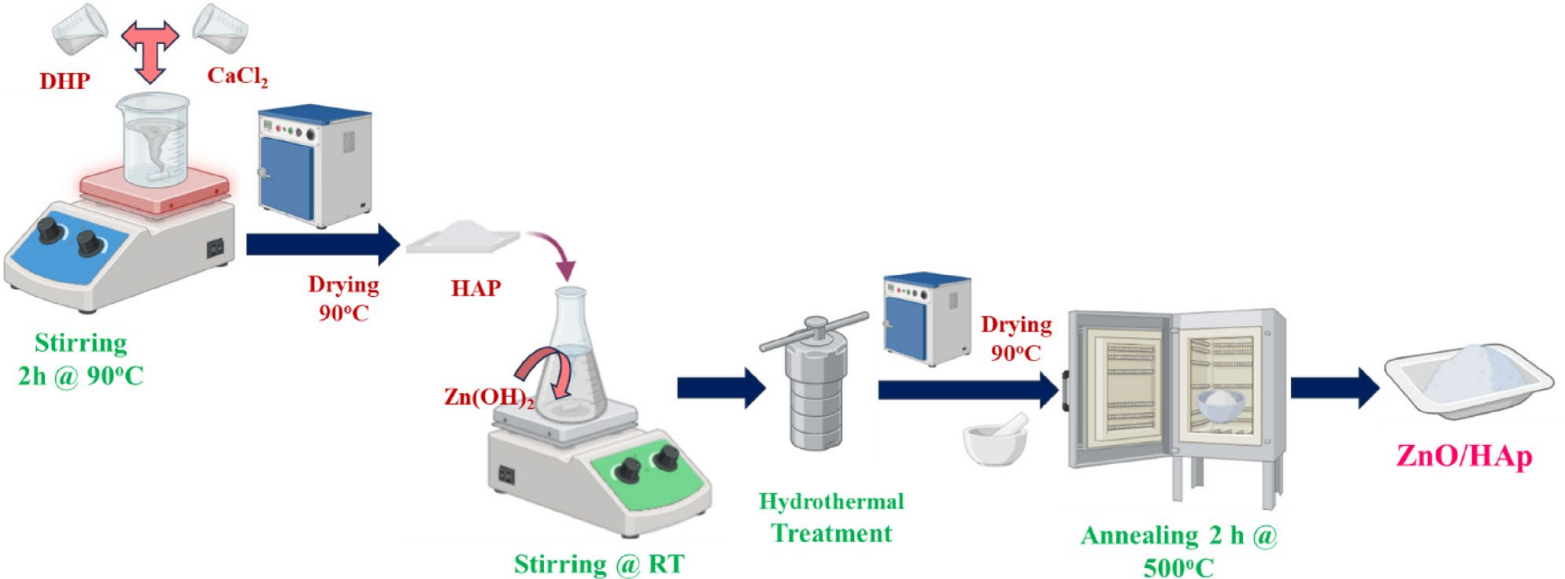
Flow diagram of synthesizing the ZnO@HAp photocatalyst.

100 ml of 0.1 M Zn(Ac)_2_·2H_2_O solution was added dropwise to 100 ml of 0.1 M NaOH solution contained in a 400 mL beaker with constant vigorous stirring for about 10 min at room temperature until an amilky mixture of Zn(OH)_2_ is obtained. Then, 500 mg of synthesized Hydroxyapatite nanoparticles was added to this Zn(OH)_2_ precipitate suspension with constant stirring in a magnetic stirrer and kept in an autoclave and heated for 8 h at 150 °C. After being filtered and dried in an oven set at 90 °C for the whole night, the combined precipitate suspension was calcined in a muffle furnace at 500 °C for 2 h. The synthesis of ZnO@Hap is depicted in Scheme 1. Then, theobtained ZnO@Hapwas groundto a fine powder.

### Characterization techniques

The prepared materials were characterized by XRD, FT-IR, SEM, TEM, and BET techniques. X-ray diffraction patterns (XRD) were investigated using a Bruker-D8 Advance ECO diffractometer. The functional group of photocatalysts was confirmed by Fourier transform infrared spectroscopic analysis (FT-IR) using an IR Tracer-100 FT-IR spectrophotometer. UV–Vis DRS was recorded on a Shimadzu UV-2900 at room temperature in the 200–800 nm range using BaSO_4_ as reference. Morphological analysis of photocatalysts was done by Scanning Electron Microscope (SEM) using EVO18-CARL ZEISS. Transmission electron microscopy (TEM) images were taken using the Tecnai G2 20 Twin TEM instrument. Nitrogen adsorption-desorption studies were carried out using a Quanto chrome, AutosorbiQ-Chemi instrument at 77 K. Before adsorption, the samples were out-gassed at 250 °C for 5 h. The XPS survey spectrum was recorded between 0 and 1300 eV with reference to the C 1s peak at 284.8 eV.

### Experimental procedure for photodegradation

The photocatalytic activity of both zinc oxide and ZnO@HAp nanocomposite photocatalysts was analyzed with an experiment of photodegradation of methylene blue. The Multi-lamp Photoreactor (Heber Multi-lamp Photo Reactor Model: HML-MP88) was used with a light source emitting 365 nm UV-A irradiation. 10 ppm methylene blue aqueous solution was used in the photodegradation experiment. To achieve the adsorption equilibrium, 50 mg of ZnO@HAp composite and 50 ml of methylene blue (10 ppm) were combined, and the mixture was continuously stirred for 30 min in the dark. After that, the solution was exposed to UV light in a photo reactor, and samples were taken at various time intervals. After the UV irradiation, the samples were analyzed for methylene blue concentration by measuring their absorbance at 664 nm using a UV spectrometer. Photodegradation of MB with ZnO catalyst was also carried out using the same procedure. A similar method was used for the degradation of ciprofloxacin (10 ppm) and sludge wastewater.

## Results and discussion

### XRD analysis

**Fig. 1:**
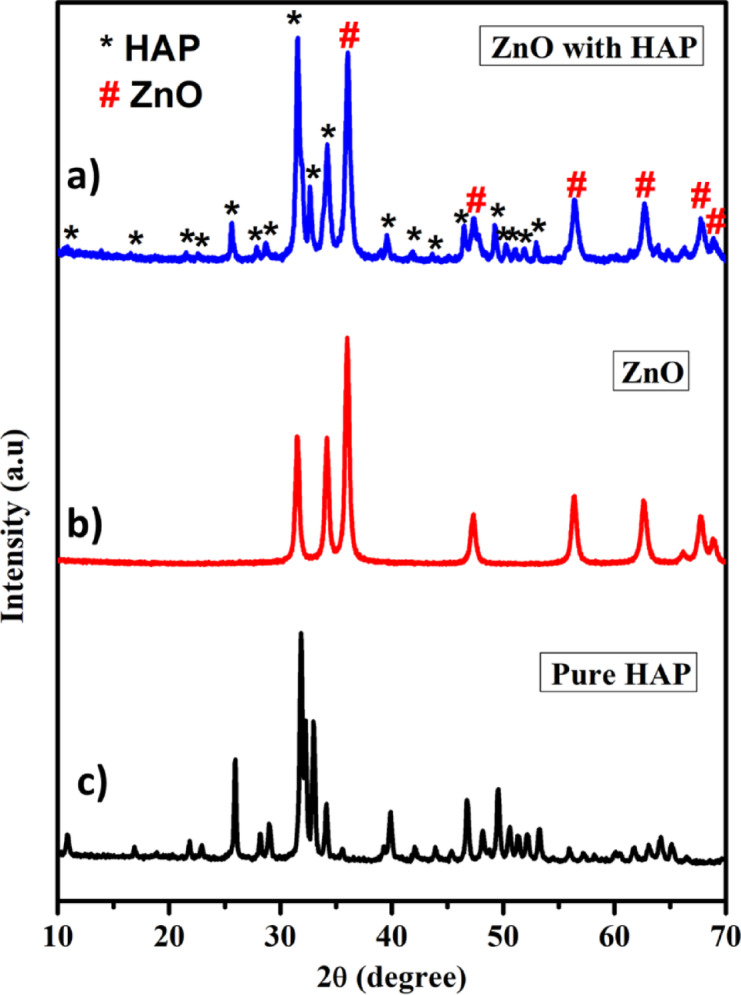
XRD patterns of photocatalysts.

The synthesized catalyst’s crystallinity, phase, and purity were investigated using an X-ray diffractometer operating in the 10–80° range with cu kα radiation (λ = 1.5418 Å). The ZnO@HAp composite, zinc oxide nanoparticle, and pure HAp’s XRD patterns are displayed in Fig. 1a–c, respectively. Figure 1a shows that all of the diffraction peaks were in good agreement with the pure hexagonal phase of hydroxyapatite (HAp). Figure 1b demonstrates that the diffraction peaks were more intense and narrower in ZnO, and this observation shows that the prepared ZnO has a wurtzite phase with a good crystalline nature^10^. Figure 1c shows the XRD pattern displaying characteristic peaks of the hexagonal phase, corresponding to the space group P6_3_/m (ICDD 00-009-0432 file). The distinct presence of HAp is confirmed by the significant diffraction peaks seen at the (211), (112), and (300) planes. These peaks remained stable in the nanocomposite even after ZnO impregnation. This suggests that in the prepared samples, HAp is the predominant phase. Furthermore, ZnO with a hexagonal crystal structure, which belongs to the space group P63/mc (JCPDS card no. 89-1397), is detected by the XRD pattern^24^. The ZnO@HAp composite’s diffraction patterns remained constant even after it formed, indicating that the structure of the materials is sustained in the composite. The peaks at 2θ: 36.36°, 56.7°, 63.02°, and 68.09° positions correspond to zinc oxide hybridized with hydroxyapatite to form a nanocomposite^24^. The XRD pattern revealed that the result was a pure ZnO@HAp composite, as no contaminants were present in the particles.

### FTIR analysis

**Fig. 2:**
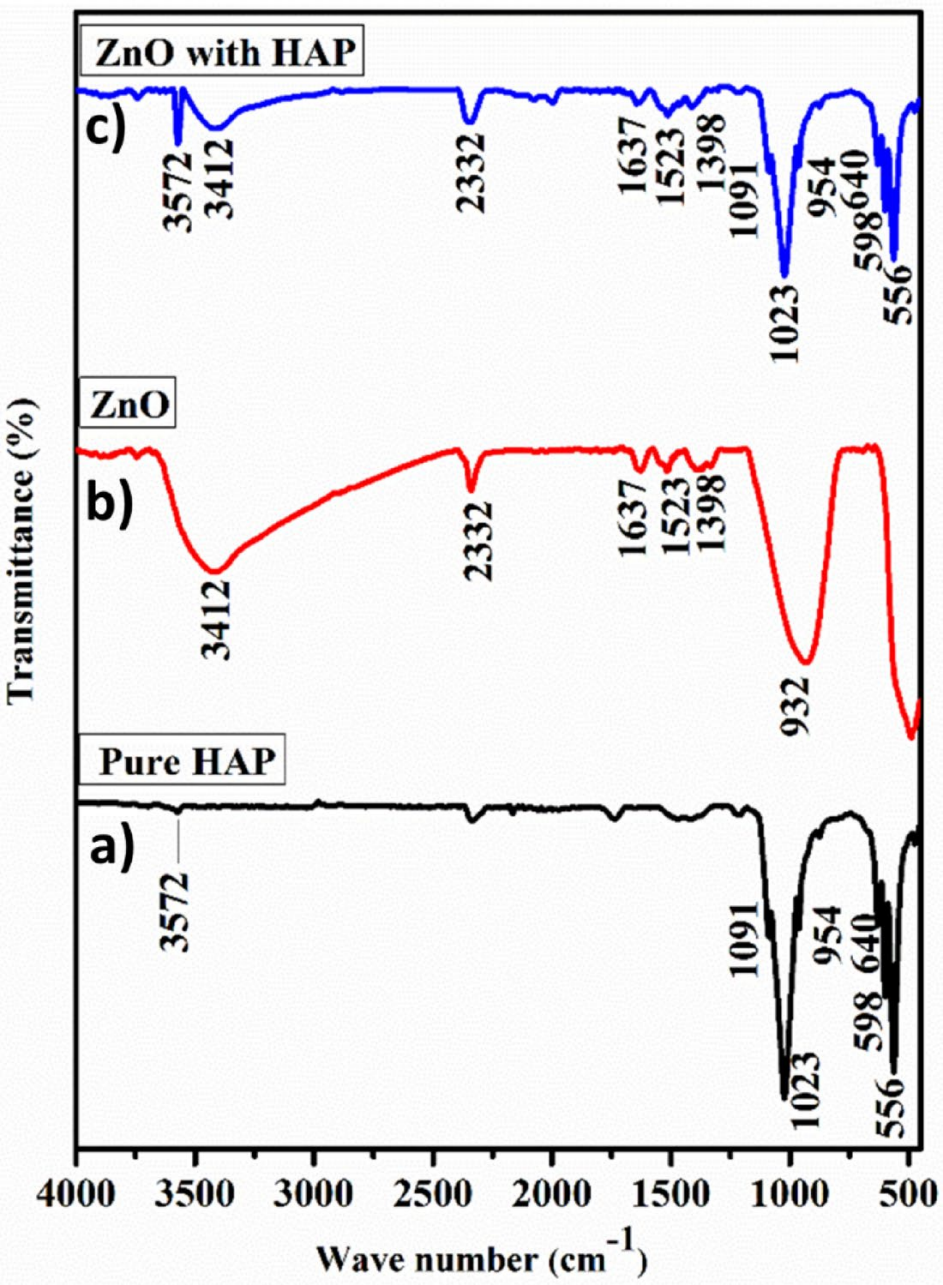
FT-IR results of photocatalysts.

Figure 2a–c presents the FT-IR spectra of the pure HAp, ZnO, and ZnO@HAp composite. The peaks at 598 and 556 cm^− 1^ in the FT-IR spectra of hydroxyapatite nanoparticles (Fig. 2a) are linked to O–P–O vibrations and represent the triply degenerate bending modes of the phosphate (PO_4_^3−^) group. The peaks at 1023 and 1091 cm^− 1^ are ascribed to the P–O bond’s asymmetric stretching vibrations. Furthermore, the vibrations of the hydroxyl (^–^OH) group found in HAp are related to the peaks observed at 3572 and 640 cm^− 1^^25^. The peaks located at 533 and 3412 cm^− 1^ in the case of zinc oxide nanoparticles are associated with the stretching vibrations of the Zn-O bond and ^−^OH. Both the distinctive peaks of zinc oxide and hydroxyapatite nanoparticles distinctive peaks were visible in the ZnO@HAp composite’s spectrum, especially at 556, 640, 1023, 2081, and 3572 cm^− 1^, which validated the composite’s formation (Fig. 2c).

### Scanning electron microscopy

**Fig. 3:**
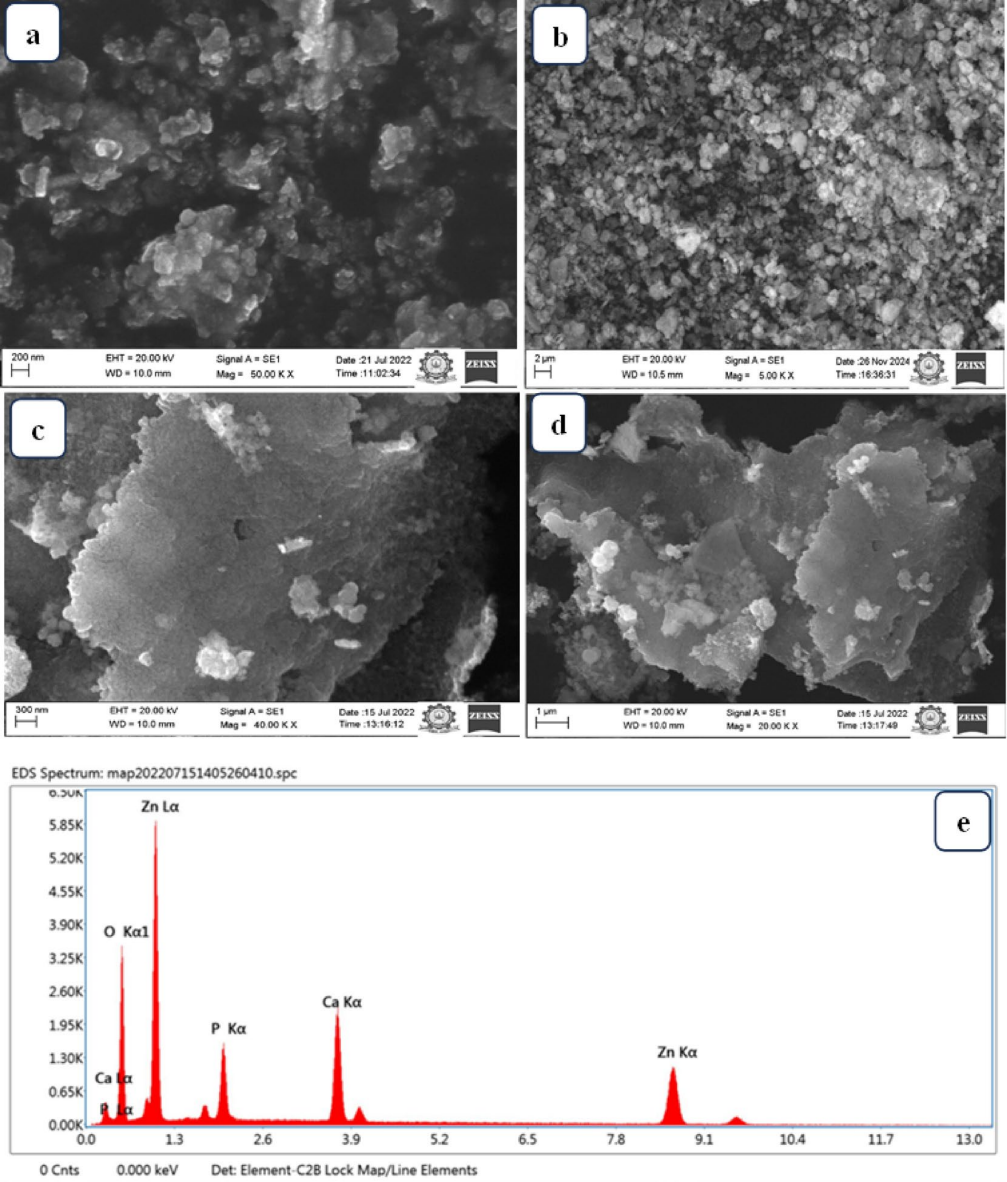
SEM-Morphological structure of (**a**) ZnO, (**b**) HAp, (**c**,**d**) ZnO@HAp, and (**e**) EDAX data of ZnO/HAp.

The SEM images were used to analyze the HAp crystal’s surface morphology. The results shown in Fig. 3a indicate that ZnO particles exhibit regular shapes with approximate sizes of 20–50 nm, and they are also agglomerated. The particle size distribution and different morphologies of platelets and flaky particles were predominant. The HAp sample’s SEM images are displayed in Fig. 3b. It illustrates the HAp’s general morphology. The HAp powder contains aggregates and granular surfaces, according to the SEM analysis. SEM images of the ZnO@HAp at two magnifications are depicted in Fig. 3c,d. We could see that the particles were agglomerated, and spherical-shaped ZnO particles were present on the surface of HAp. EDAX data of prepared material ZnO@HAp, as seen in Fig. 3e, indicate the presence of zinc, oxygen, phosphorus, and calcium in the prepared materials, confirming the formation of the composite.

### BET analysis

**Fig. 4:**
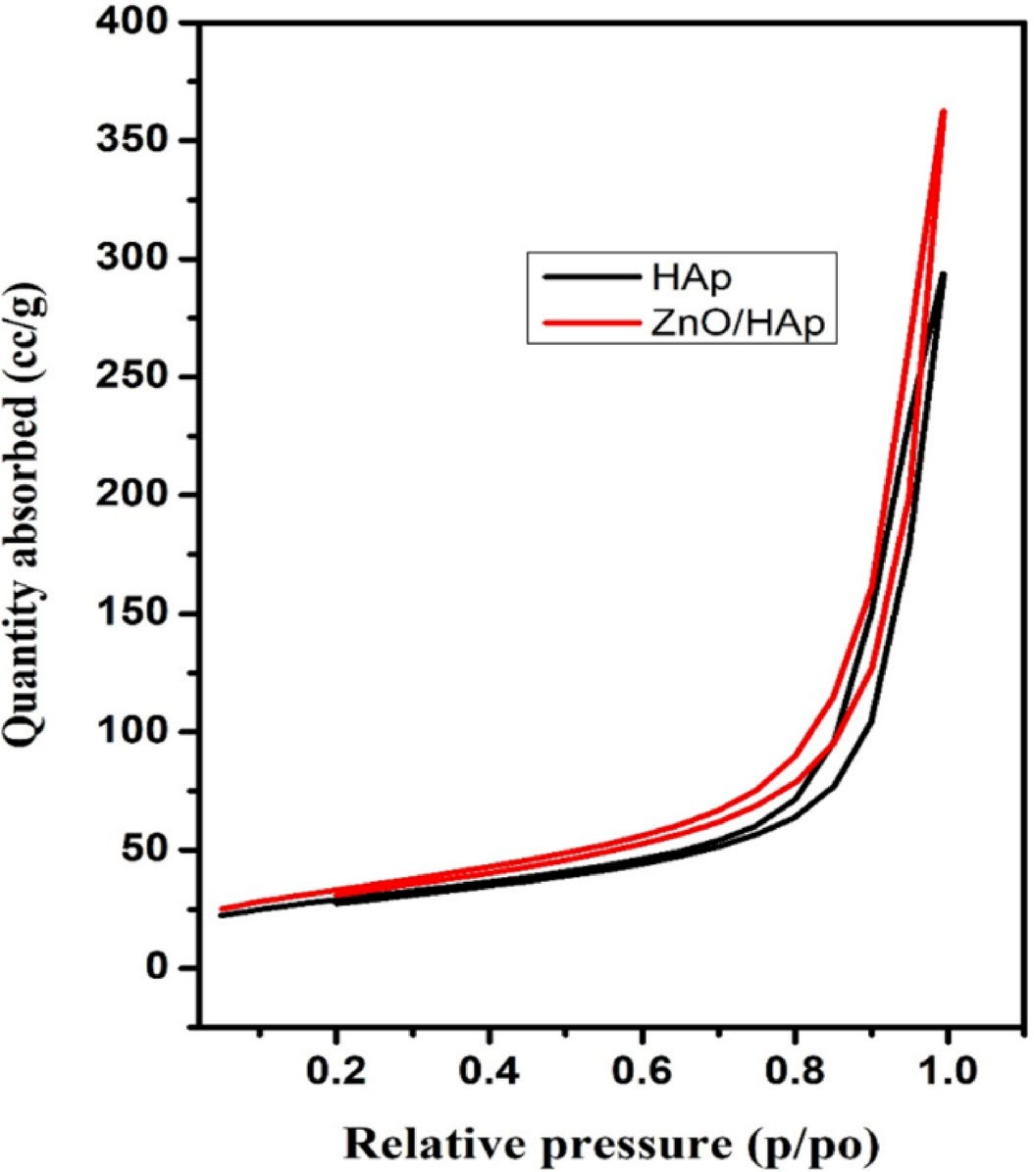
Nitrogen adsorption isotherms of the prepared materials.

The developed photocatalyst’s nitrogen adsorption and desorption isotherms are shown in Fig. 4. The presence of porous materials is indicated by the type-IV nitrogen adsorption-desorption isotherms with a noticeable hysteresis loop in the relative pressure (P/P_o_) range of 0.2 to 1.0, which are displayed by both ZnO and ZnO@HAp. In comparison to pure ZnO, the ZnO@HAp nanocomposite’s BET study showed a noticeably larger surface area. With a total pore volume of 0.0929 cm^3^/g, micropore volume of 0.004 cm^3^/g, micropore surface area of 9.179 m^2^/g, and exterior surface area of 87.096 m^2^/g, the calculated specific surface area was 110.124 m^2^/g. These findings demonstrate that ZnO@HAp has a significantly higher BET surface area (110.124 m^2^/g) than pure ZnO (30.2 m^2^/g) and a well-developed porous structure. The higher surface area of ZnO@HAp enhances its adsorption characteristics, consequently increasing photocatalytic activity.

### TEM analysis

**Fig. 5:**
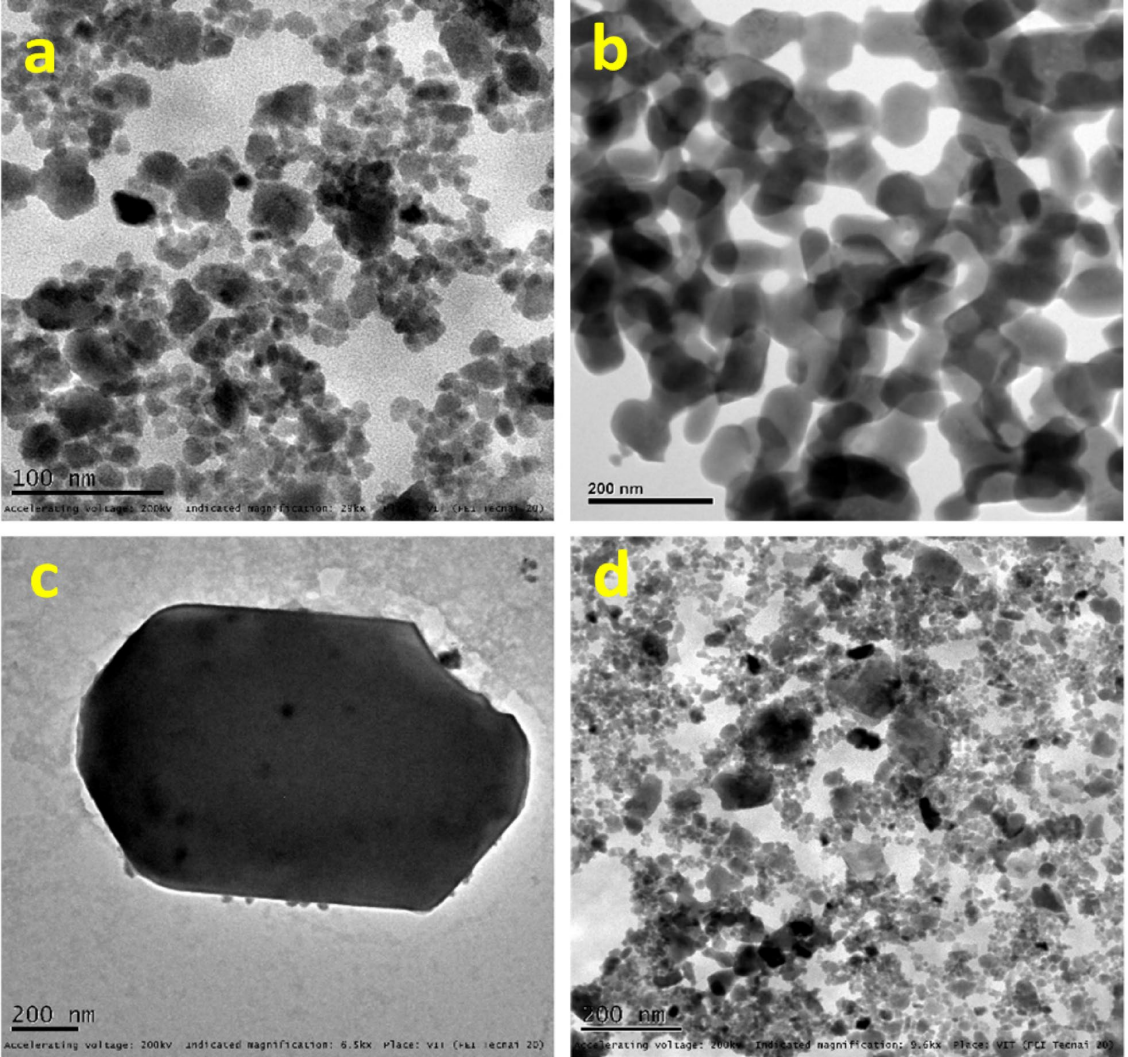
TEM images of HAp (**a**,**b**) and ZnO@HAp (**c**,**d**).

TEM research was used to further investigate the synthesized photocatalyst’s structural and morphological properties. The presence of particles in various forms, including spherical and hexagonal structures of pure HAp, is visible in the TEM images of the material (Fig. 5a,b). The findings of the SEM are in agreement with these observations. Moreover, ZnO@HAp nanoparticles are comparatively monodispersed, with an average particle size ranging from roughly 25 to 40 nm, according to the TEM images of ZnO@HAp nanocomposites (Fig. 5c,d).

### XPS analysis

**Fig. 6:**
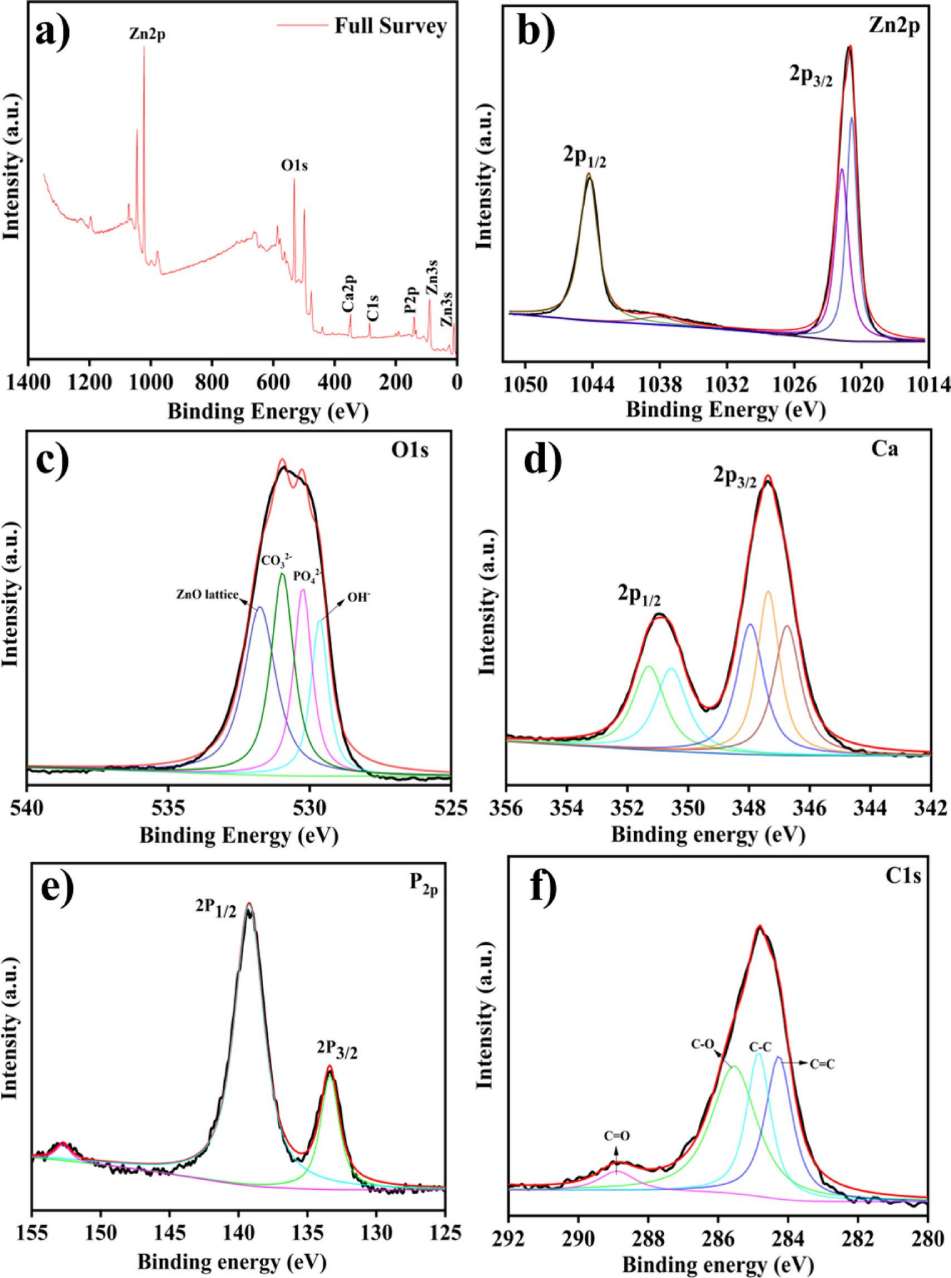
XPS spectra of ZnO/HAp (**a**) Full survey, (**b**) Zn 2p, (**c**) O 1s, (**d**) Ca 2p, (**e**) P 2p, and (**f**) C 1s.

X-ray Photoelectron Spectroscopy (XPS) was performed to investigate the surface chemical composition of the as-synthesized ZnO@HAp composite. Figure 6 displays the binding energies corresponding to the elemental states of O, Ca, P, Zn, and C. The survey spectrum (Fig. 6a) confirms the presence of Zn, O, Ca, P, and C through their characteristic peaks. In particular, the Zn 2p region (Fig. 6b) shows two distinct peaks at 1021.7 eV and 1044.1 eV, which correspond to Zn 2p_3/2_ and Zn 2p_1/2_, respectively, indicating the formation of Zn–O bonds in the composite^26^. The O 1s spectrum (Fig. 6c) was deconvoluted into four distinct peaks located at 529.6 eV, 530.2 eV, 530.9 eV, and 531.7 eV. These peaks can be attributed to lattice oxygen in ZnO, carbonate (CO_3_^2−^), phosphate (PO_4_^3−^), and hydroxyl groups (OH^−^), respectively^27^. Figure 6d shows two major peaks at 347.9 eV and 351.3 eV, corresponding to the Ca 2p_3/2_ and Ca 2p_1/2_ core levels, which confirm the presence of calcium in the composite. The Ca 2p_3/2_ peak originated from calcium bonding and its interaction with adsorbed CO_3_^2−^ in HAp, while the Ca 2p_1/2_ peak corresponded to metallic calcium^28^. The P 2p spectrum (Fig. 6e) displays two significant peaks at 133.3 eV and 139.2 eV, assigned to P 2p_3/2_ and P 2p_1/2_, respectively, indicating the presence of P–O bonds in the HAp structure^29^. Finally, Fig. 6f presents the C 1s and ZnO region of the XPS spectrum. The C 1s peak appears at 284.8 eV and is typically used as a reference for charge correction. Notably, the binding energy of the C 1s peak remains unchanged after the partial substitution of Ca^2+^ ions with Zn^2+^ ions, and consistent with previous reports^30^.

### Photocatalytic activity and effect of catalyst amount

**Fig. 7:**
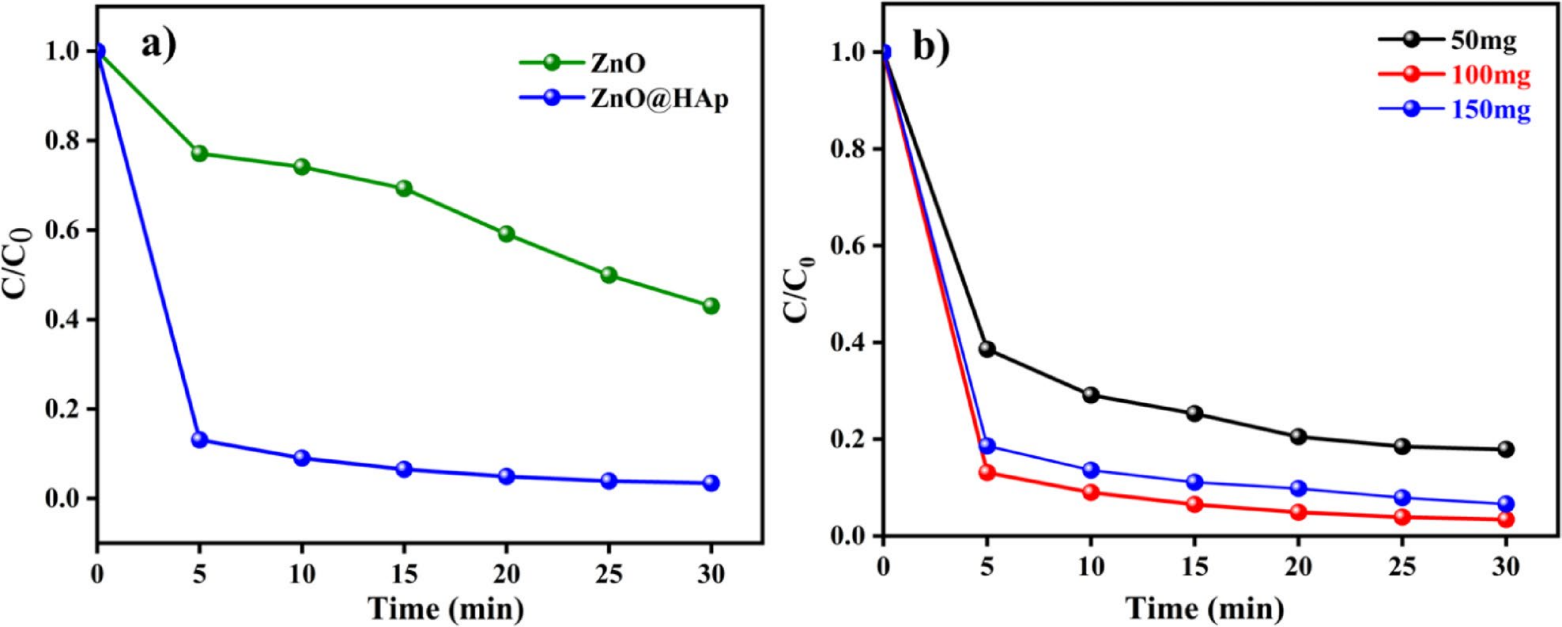
(**a**) Methylene blue (10 ppm/50 mL) degradation with ZnO, ZnO@HAp (50 mg), pH 7, and (**b**) catalyst dosage of ZnO@Hapon MB degradation.

The photocatalytic degradation of methylene blue(MB) dye under UV irradiation (365 nm) was investigated using ZnO and ZnO@HAp catalysts. Even after 30 min, we don’t see the complete degradation of methylene blue dye. We have achieved a degradation efficiency of less than 40%. Hence, the ZnO alone may not be useful for industrial dye degradation. In general, HAp does not degrade organic dye due to its large energy band gap, which might be the reason why the photocatalytic efficiency has not been improved over HAp. As seen in Fig. 7a, the methylene blue undergoes almost90% degradation in 5 min with ZnO@HAp.

**Fig. 8:**
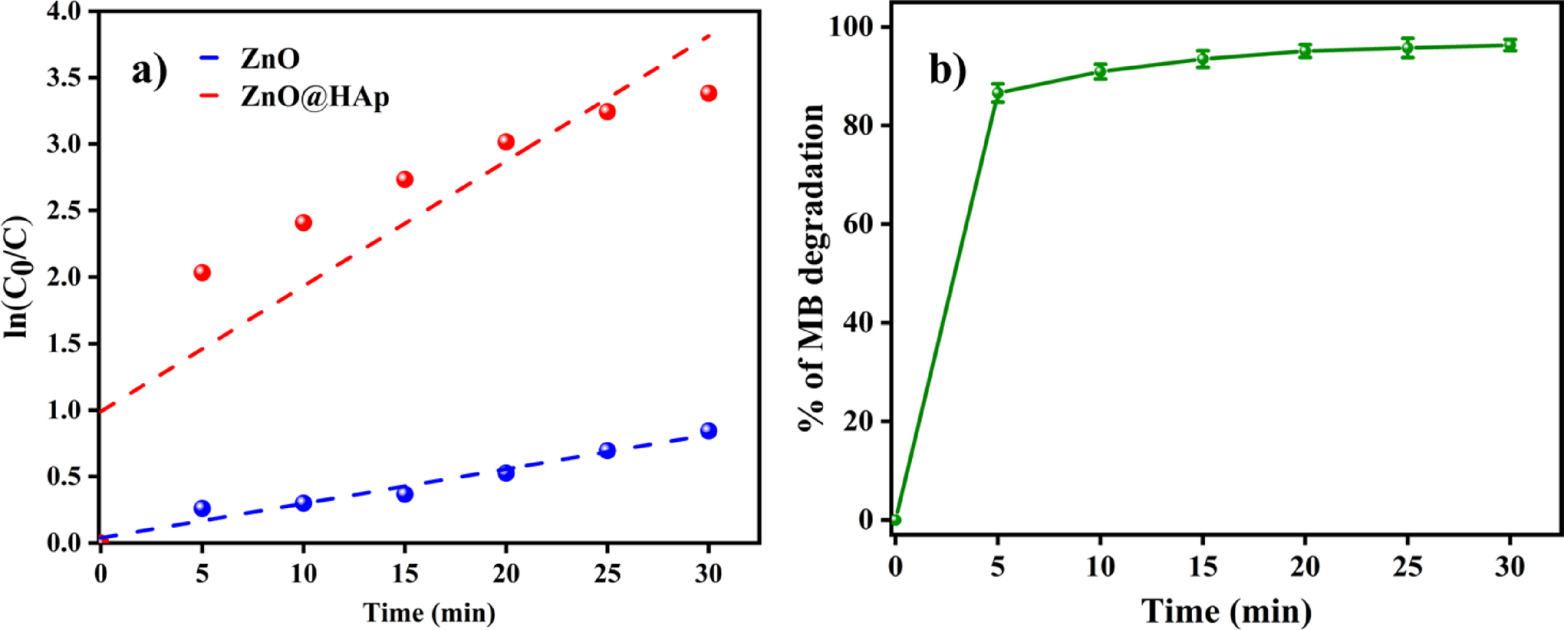
(**a**) Methylene blue degradation kinetics of ZnO, ZnO@HAp, and 0 (**b**) Error bar of ZnO@Hapon MB degradation.

Photocatalytic studies were conducted using varying doses of the catalyst, ranging from 50 to 150 mg, to determine the optimal dosage of ZnO@HAp. Figure 7b illustrates how the availability of additional active sites and higher production of hydroxyl radicals caused the degradation rate of methylene blue to increase with catalyst loading up to 100 mg. However, efficiency decreased when the catalyst amount exceeded 100 mg. This was probably because increased turbidity and light scattering made it more difficult for photons to penetrate. Therefore, 100 mg was found to be the optimal dosage of ZnO@HAp for efficient methylene blue degradation. As shown in Fig. 8a, the ZnO@HAp (k = 0.0281 min^− 1^) catalyst exhibits a pseudo-first-order rate for MB degradation that is higher than pure ZnO (k = 0.1127 min^− 1^), indicating enhanced photocatalytic efficiency. Error bars (Fig. 8b) represent standard deviations from triplicate experiments, confirming the reliability of the degradation performance.

**Fig. 9:**
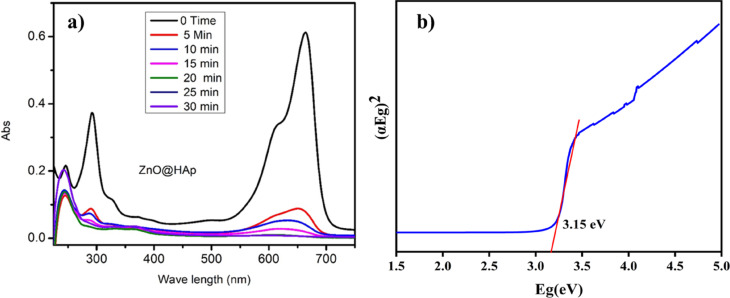
(**a**) Methylene blue (10 ppm/50 mL) degradation absorption spectra with ZnO@HAp (50 mg), pH 7, and (**b**) Tauc plot of prepared ZnO@HAp.

The absorption spectra data of methylene blue over ZnO@HAp with different irradiation time intervals are shown in Fig. 9a. The optical energy band gap of ZnO@HAp, calculated using a Tauc plot, was found to be 3.15 eV (Fig. 9b), which is lower than the bandgap energies reported for ZnO (3.26) and HAp (4.2 eV)^31,32^. The optical features of ZnO@Hap nanocomposite cause charge transfer from ZnO to HAp and reduce charge carrier recombination in zinc oxide. As a result, hydroxyl radicals are produced on composite surfaces, accelerating the photodegradation process. The ZnO@HAp photocatalyst is found to be much more efficient than ZnO and HAp. The higher efficiency of ZnO@HAp might be due to the strong interaction between ZnO and HAp. Additionally, the SEM analysis reveals that ZnO nanoparticles are located on the surfaces and within the pores of the HAp. This could be responsible for the increased catalytic activity of the ZnO@HAp composite. No degradation was observed when the reaction proceeded without a catalyst. Therefore, compared to pure ZnO, ZnO@HAp’s greater specific surface area and porous architecture improve its photocatalytic efficacy toward organic pollutants. Results confirm that ZnO was evenly combined with the HAp matrix in the ZnO@HAp nanocomposite, which exhibits a high specific surface area relative to individual ZnO and HAp. The amount of the catalyst and recyclability are important aspects of photodegradation.

**Fig. 10:**
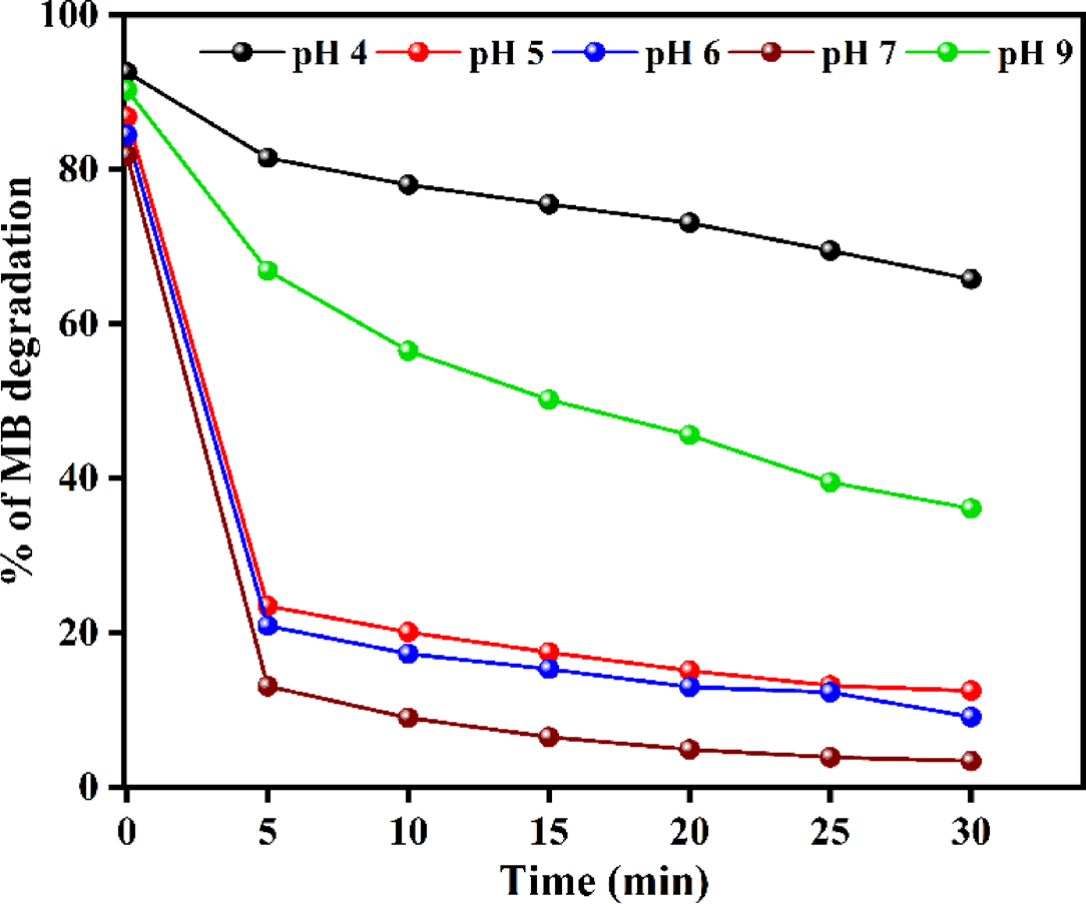
Effect of initial solution pH on MB degradation.

The pH of the solution plays a crucial role in the photocatalytic degradation process. Investigating the effect of pH in the range of 4–9 (Fig. 10) revealed that the maximum degradation efficiency of 96.4% was achieved at a neutral pH of 7 within 30 min. The reduced effectiveness below this pH is attributed to the partial solubility of ZnO in the ZnO@HApcatalyst under acidic environments.

Since the catalyst surface becomes more negatively charged and the electrostatic affinity with anionic dye species is weakened, the degradation efficiency decreases above pH 7, resulting from reduced adsorption of dye molecules. For pH levels of 4, 5, 6, 7, and 9, the corresponding adsorption measurements at equilibrium were 7.4%, 13.2%, 15.6%, 19.1%, and 9.7%, respectively. The highest adsorption was observed at a pH of 7. The ideal pH for methylene blue mineralization is 7, as confirmed by the strong correlation between adsorption and degradation efficiency.

### Catalyst recyclability

**Fig. 11:**
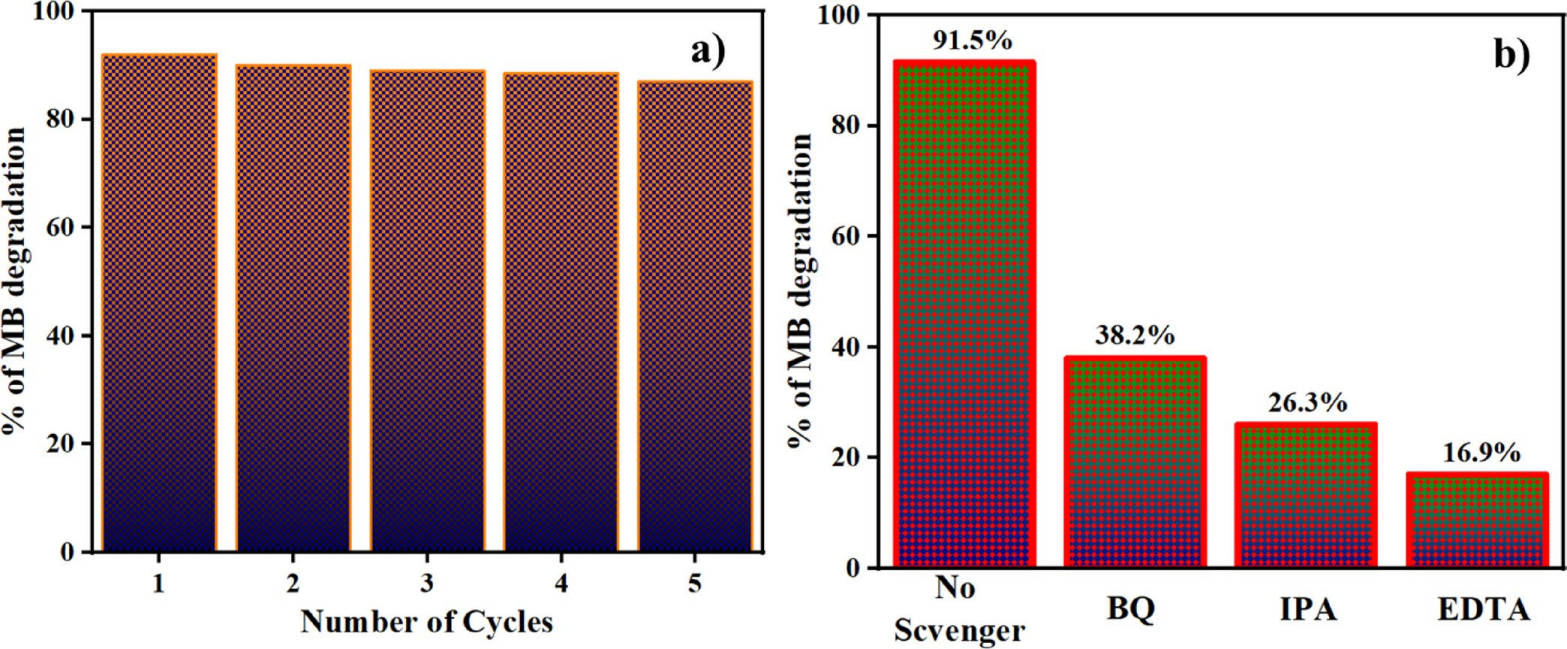
(**a**) Reusability of ZnO@HAp, and (**b**) Scavenger test of ZnO@HAp catalyst.

Recycling studies of ZnO@HAp for the degradation of methylene blue dye under optimized reaction conditions were performed over 5 cycles, and the results are shown in Fig. 11a. It is important to note that even after the five consecutive recycles, the photocatalytic efficiency is not much affected. In addition, the recycled catalyst was separated and dried, and then analyzed by powder XRD. The XRD pattern of the recycled photocatalyst is shown in Figure S1 (*Please see supporting information*). We found no significant changes even after five recycles. These results clearly indicate the stable nature of the catalyst.

### Scavenger test

**Fig. 12:**
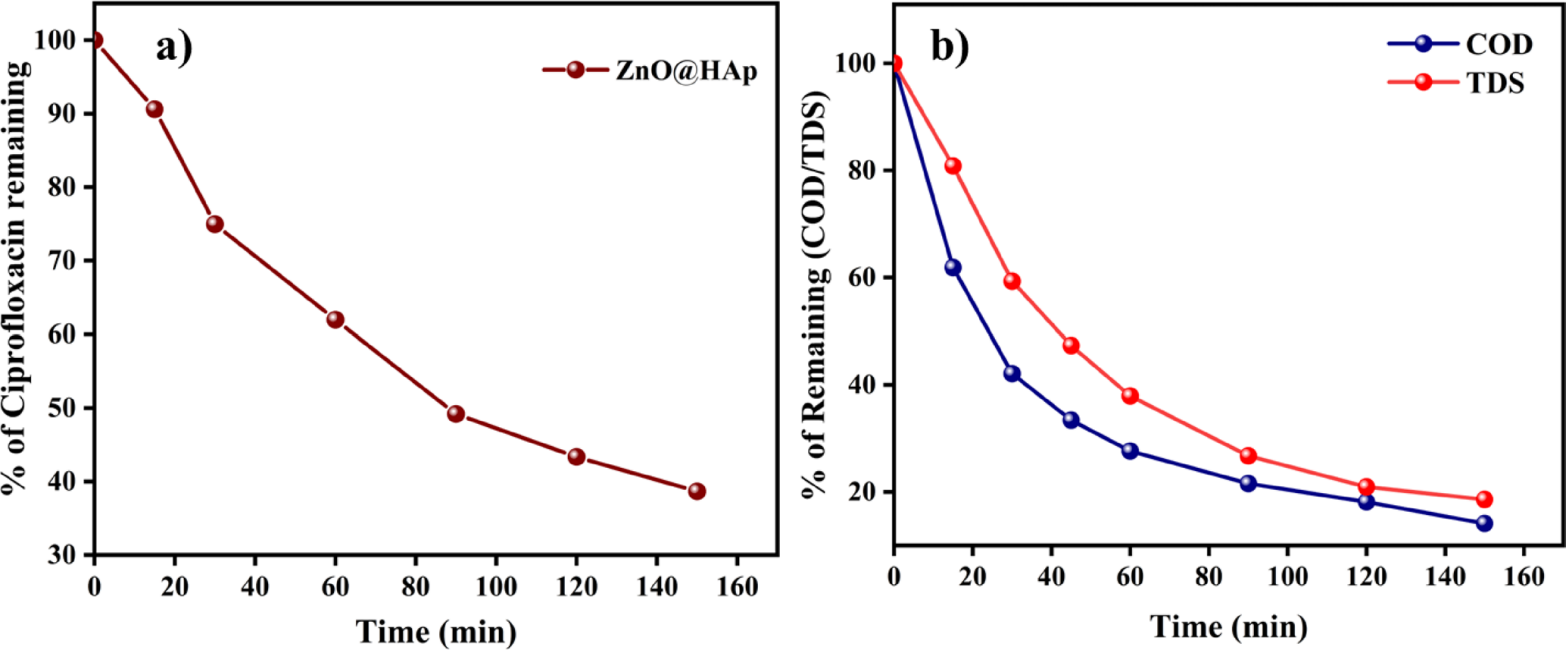
Ciprofloxacin degradation (**a**) Sludge wastewater treatment of ZnO@HAp (**b**).

The process of photocatalytic degradation occurs through the generation of radicals with light. If radical scavengers are introduced, they can reduce the concentration of radicals and impact the rate of photodegradation. To identify the active radical species responsible for photocatalytic activity, four scavengers were used during the degradation of the methylene blue dye. The ethylenediaminetetraacetic acid (EDTA, 10 ppm), p-benzoquinone (BQ, 10 ppm), isopropyl alcohol (IPA, 10 ppm), and ethanol were used as h^+^, superoxide radical (^·^O^2−^), and hydroxyl radical (^·^OH) scavengers, respectively. The results indicate that the EDTA scavenger, which removes holes (h^+^) from the system, has a significant impact on the assessment of photocatalytic activity (Fig. 11b). As per the scavenger test, holes (h^+^) are considered the principal active species in photocatalytic degradation. As shown in Fig. 11b, the addition of BQ caused the degrading efficiency to decrease moderately by 53%, while the addition of IPA and EDTA, at the same volume and concentration, caused a considerable suppression of roughly 65% and 75%, respectively. Therefore, hydroxyl radicals (^·^OH) and holes (h^+^)play the dominant role in the photodegradation of methylene blue; however, h⁺ plays a more significant role in the ZnO@HAp photocatalytic system than ·OH. Additionally, the efficiency of the catalyst was investigated for the degradation of the drug ciprofloxacin and in the treatment of domestic wastewater. Ciprofloxacin degradation is shown in Fig. 12a. About 63% of degradation occurred in 150 min of irradiation. The kinetic plot for ciprofloxacin degradation is shown in Figure S2 (*Please see supporting information*). In domestic wastewater treatment, both COD and TDS were measured to monitor the degradation process. Changes in COD and TDS are displayed in Fig. 12b. About 90% COD reduction was observed in 150 min of irradiation. These results confirm the efficiency of the catalyst in the treatment of domestic and pharmaceutical industrial wastewater.

**Fig. 13:**
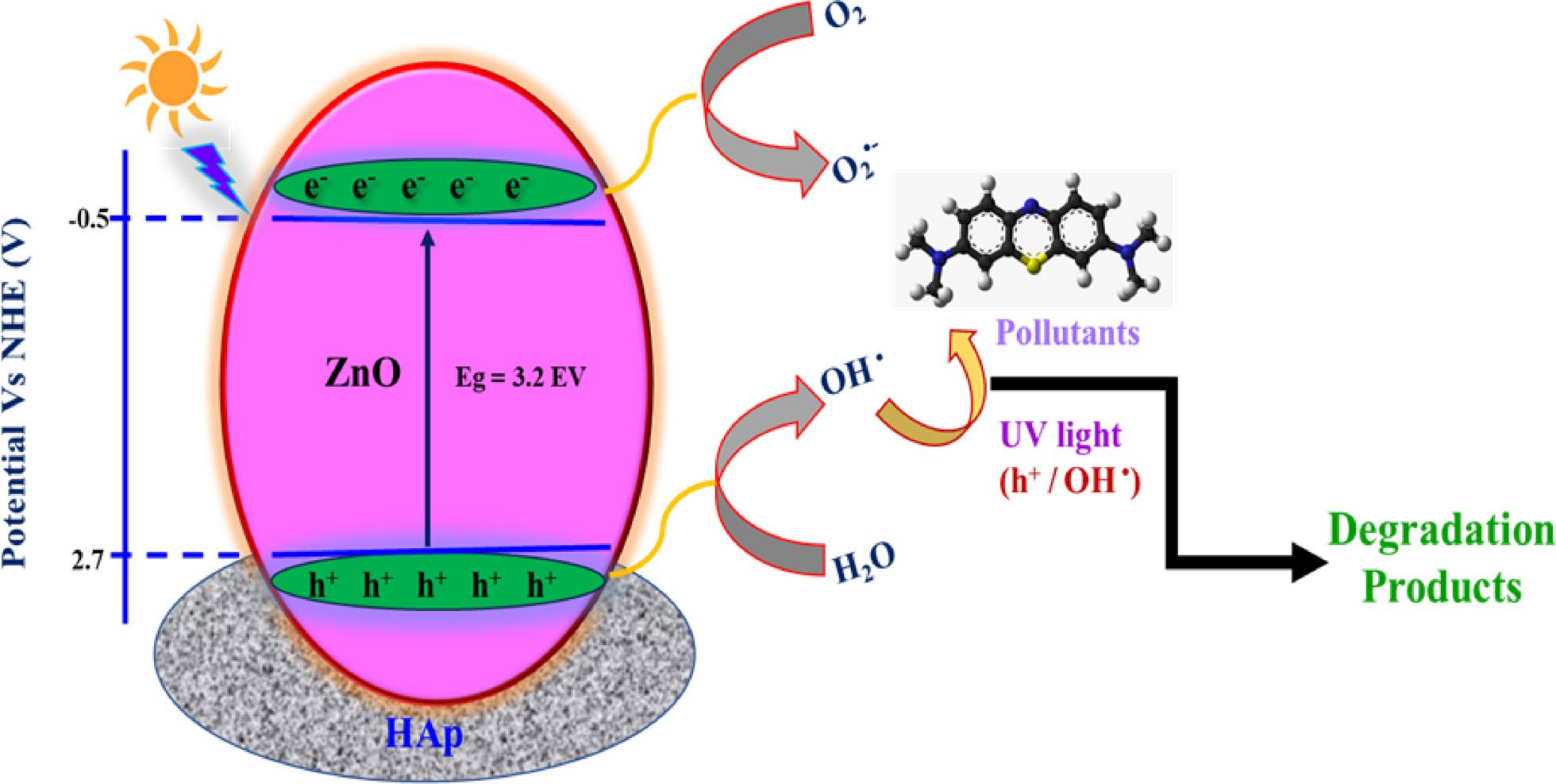
Degradation mechanism of ZnO@HApcatalyst.

Based on the scavenger test, a proposed mechanism of degradation is shown in Fig. 13. The concentration of hydroxyl groups on the catalyst surface is significantly increased by the addition of HAp to ZnO. Pollutant breakdown is facilitated by the increased number of hydroxyl groups, which act as efficient trapping sites for photogenerated holes^33^. Additionally, the presence of HAp increases the number of molecular oxygen adsorption sites, facilitating the trapping of photogenerated electrons and producing more hydroxyl radicals. Furthermore, the high adsorption characteristics of HAp enable it to adsorb the pollutant molecules and release them synergistically for degradation by ZnO. Higher surface area of ZnO@HAp (110.124 m^2^/g) than pure ZnO (30.2 m^2^/g causes an increased adsorption of pollutants and therefore, as compared to pure ZnO, the ZnO@HAp nanocomposite demonstrated better photocatalytic activity.

The photocatalytic performance of ZnO@HAp for MB degradation is compared with earlier results in Table 1 to demonstrate its effectiveness. ZnO@HAp demonstrated higher activity and reusability, achieving 96% degradation in 30 min and maintaining 87% efficiency after five cycles, whereas many other reported catalysts required longer timeframes^34–37^.

**Table 1:** Comparison of the photocatalytic efficiency of some photocatalysts toward MB degradation.

S. no.	Catalysts	Dosage of catalysts and Methylene Blue dye (mg)	Irradiation time (min)	Efficiency (%)	Refs.
1	CdS/BiVO_4_/g-C_3_N_4_	10/20	120	94.5	^34^
2	Gd and Y co-doped BiVO_4_	100/10	90	94	^35^
3	MgO/MnO_2_	75/10	60	87	^36^
4	CuO and NiO-infused MnO_2_	10/10	100	89.05	^37^
5	ZnO/HAp	40/10	30	96.5	In this work

## Conclusion

In this study, ZnO@HAp nanocomposite was prepared using a simple precipitation method. SEM images reveal the spherical-shaped ZnO particles on the surface of HAp. The TEM pictures revealed that the synthesized ZnO@HApNCs were quite monodispersed, with an average diameter of approximately 25–40 nm. Methylene blue undergoes almost 90% degradation in 5 min with ZnO@HAp. About 63% in ciprofloxacin(150 min) degradation and 88% COD reduction in domestic sludge were observed in 150 min of irradiation. We agree that the ZnO@HAp photocatalyst we prepared, which has a greater specific surface area, provides better photodegradation performance for organic pollutants and wastewater treatment. The higher efficiency and stability of the prepared ZnO@HAp composite over ZnO and pure HAp make it suitable for any type of effluent treatment.

## Supplementary Information

Below is the link to the electronic supplementary material.

Supplementary Material 1

## Data Availability

All data generated or analysed during this study are included in this article.
