# Supplementary material for: Enhanced efficiency of zinc oxide hydroxyapatite nanocomposite in photodegradation of methylene blue, ciprofloxacin, and in wastewater treatment
Source: Sci Rep. 2025 Aug 29;15:31819. doi: 10.1038/s41598-025-14310-7 (PMC12397344; doi:10.1038/s41598-025-14310-7)
Supplement: Supplementary file 1 — Supplementary Material 1 [file 41598_2025_14310_MOESM1_ESM.docx]

# SUPPLEMENTARY MATERIAL

# Enhanced efficiency of zinc oxide hydroxyapatite nanocomposite in the photodegradation of methylene blue, ciprofloxacin and in wastewater treatment

**Table of content**

| Figure No. | Figure caption | Page |
| --- | --- | --- |
| Figure S1 | XRD pattern of ZnO@HAp post-degradation. | **S2** |
| Figure S2 | Kinetics plot of ciprofloxacin degradation with ZnO@HAp. | **S2** |

**
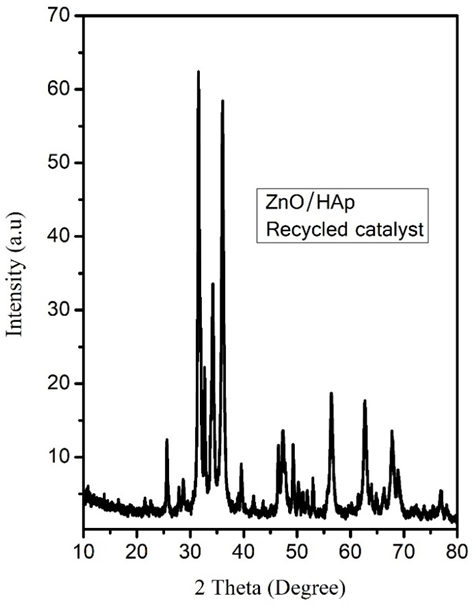
**

**Figure S1.** XRD pattern of ZnO@Hap post-degradation


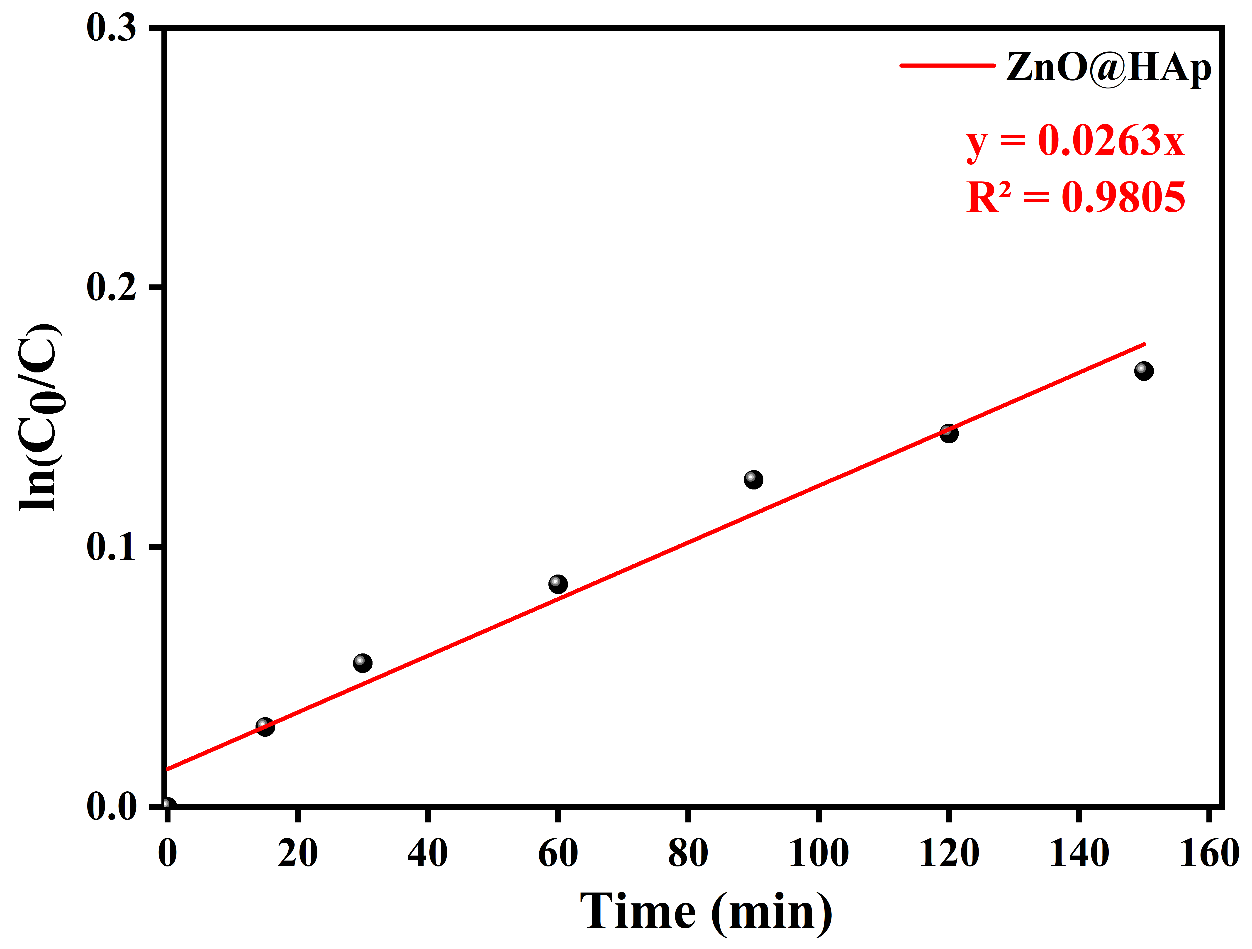


**Figure S2.** Kinetics plot of Ciprofloxacin degradation with ZnO@HAp

**Figure S2:** Effect of catalyst weight on degradation of methylene blue over ZnO@HAp. Condition: Catalyst amount 50 mg, 10 ppm solution,

(C)
